# The Esc(1-21)-1c Antimicrobial Peptide Inhibits a Specific Transcriptional Activator of the MexAB–OprM Efflux Pump in *P. aeruginosa*

**DOI:** 10.3390/ijms26209940

**Published:** 2025-10-13

**Authors:** Carolina Canè, Bruno Casciaro, Carlo Vetrano, Lidia Tammaro, Chiara Platella, Domenica Musumeci, Maria Luisa Mangoni, Angela Duilio, Angela Di Somma

**Affiliations:** 1 Department of Molecular Medicine and Medical Biotechnology, University of Naples “Federico II”, 80131 Naples, Italy; carolina.cane@unina.it; 2 Laboratory Affiliated to Pasteur Italia-Fondazione Cenci Bolognetti, Department of Biochemical Sciences, Sapienza University of Rome, 00185 Rome, Italy; bruno.casciaro@uniroma1.it (B.C.); carlo.vetrano@uniroma1.it (C.V.); marialuisa.mangoni@uniroma1.it (M.L.M.); 3 CEINGE Biotecnologie Avanzate, 80145 Naples, Italy; tammarol@ceinge.unina.it; 4 Department of Chemical Sciences, University of Naples “Federico II”, 80126 Naples, Italy; chiara.platella@unina.it (C.P.); domenica.musumeci@unina.it (D.M.); angela.duilio@unina.it (A.D.); 5 Institute of Biostructure and Bioimaging (IBB)-CNR, 80145 Naples, Italy; 6 National Institute of Biostructure and Biosystems (INBB), 00165 Rome, Italy

**Keywords:** Esc (1-21)-1c, Q9I5H3 regulatory protein, MexAB-OprM efflux pump

## Abstract

The emergence of multidrug-resistant *Pseudomonas aeruginosa* strains is increasingly becoming a critical threat to global health. Among the resistance mechanisms, the MexAB–OprM efflux pump confers *P. aeruginosa* with an efficient method to export a broad spectrum of antibiotics. The antimicrobial peptide Esc (1-21)-1c was shown to downregulate this efflux system, though its mechanism of action has not been unveiled thus far. Here, we employed a combination of molecular modeling and experimental methods to investigate the precise peptide inhibitory mechanism. Functional proteomic experiments revealed the *P. aeruginosa* protein Q9I5H3, homologous to *E. coli* QseB, as a putative key target of Esc(1-21)-1c. Molecular docking predicted stable peptide–protein interactions, which were experimentally validated through fluorescence spectroscopy. Furthermore, electrophoretic mobility shift assays demonstrated that Q9I5H3 specifically binds the MexAB–OprM promoter and that Esc(1-21)-1c competitively inhibits this interaction in a dose-dependent manner. These findings reveal a previously uncharacterized regulatory pathway for efflux pump control and highlight Q9I5H3 as a promising therapeutic target against multidrug-resistant *P. aeruginosa*.

## 1. Introduction

Nowadays, the growing surge of microorganisms showing antibiotic resistance is a major global health threat, leading to increased mortality and contributing to the spread of resistant infections [1]. Indeed, the number of microbial infections that are unresponsive to current antibiotics is increasing, while the number of new approved antibiotics is not keeping up [2,3]. According to the latest World Health Organization reports, this trend could lead to approximately 10 million deaths annually worldwide by 2050, if no action is taken [4].

*Pseudomonas aeruginosa* is one of the most dangerous bacterial pathogens responsible for severe infections, due to its ability to easily develop resistance to antibiotics and to form biofilm communities. Within biofilms, bacterial cells enter into a dormant, metabolically inactive state and are physically protected from drugs by their self-produced extracellular matrix [5,6]. The intrinsic and acquired resistance mechanisms of *P. aeruginosa*, including the expression of efflux pumps such as the MexAB–OprM system, make this pathogen particularly challenging to treat and an important target for the development of novel therapeutic approaches [7]. Given the limitations of conventional antibiotics, the search for new alternatives to traditional drugs has become highly pressing. Cationic antimicrobial peptides (AMPs) represent promising molecules showing a general membrane-perturbing mechanism of bactericidal activity, which restricts the induction of resistance and suggests AMPs as putative next-generation therapeutics in the biomedical field [8,9]. Moreover, at sublethal doses, AMPs have been found to display additional mechanism(s) involving interaction with intracellular targets and, hence, defined as “dirty drugs” [10,11]. This multitarget approach makes AMPs particularly attractive as they can simultaneously address multiple bacterial resistance mechanisms.

In the past years, we characterized the biological properties of a derivative of the frog-skin AMP esculentin-1a, namely Esc(1-21)-1c of sequence GIFSKLAGKKIKN^d^LLI^d^SGLKG-NH2 containing two D-amino acids, i.e., Leu14 and Ser17. At levels below the minimal growth inhibitory concentration (MIC), the peptide showed the capability to prevent *P. aeruginosa* biofilm formation and a synergic effect with the antibiotic Aztreonam in inhibiting growth and in killing this bacterium [12,13,14]. Furthermore, very recent studies have provided new insights into the global impact of Esc (1-21)-1c on *P. aeruginosa*. Differential proteomics and transcriptional analyses demonstrated that at sub-MIC dosages, Esc(1-21)-1c has a significant downregulatory effect on MexA, MexB, and OprM proteins [15] which collectively form an important efflux pump system known to have an essential function in antibiotic resistance in *P. aeruginosa* [7]. Overall, such outcome suggested that Esc(1-21)-1c is able to enhance the effectiveness of antibiotics by reducing their export thus allowing drugs accumulation inside the bacteria cells. These effects make *P. aeruginosa* more susceptible to conventional antibiotic treatments, providing a promising approach to fight infections caused by this pathogen.

Despite similar findings (inhibition of OprM and MexA genes expression) have been reported for the Trp containing peptides LHW and L12W [16], no studies have been performed to understand the mechanism underlying this process. Therefore, in-depth investigations are imperative to unravel the specific molecular mechanisms accounting for the downregulation of MexAB efflux pump by Esc(1-21)-1c. Here, we explored the intracellular mechanism of Esc(1-21)-1c at the molecular level by the identification of its putative interactors in *P. aeruginosa*. Functional proteomics approaches allowed us to identify protein PA0756, a novel transcriptional activator of the MexAB–OprM operon, as the direct interactor of the peptide. This protein belongs to a two-component system (TCS) which is known to play crucial roles in modulating gene expression in response to environmental cues such as exposure to antibiotics in Gram-negative bacteria [17]. A multidisciplinary approach involving computational studies, recombinant expression of the target protein, and molecular biology experiments confirmed previous proteomics data, eventually leading to the elucidation of the downregulation effect exerted by Esc(1-21)-1c on the MexAB–OprM operon. These data provide a solid foundation for the development of highly specific and efficient drugs targeting this protein. This is particularly relevant in the current post-pandemic era, where the growing threat of multidrug resistant (MDR) bacteria needs to be rapidly and rationally addressed [18] through rational therapeutic strategies.

## 2. Results

### 2.1. Mechanism of Action of Esc(1-21)-1c

**Figure 1:**
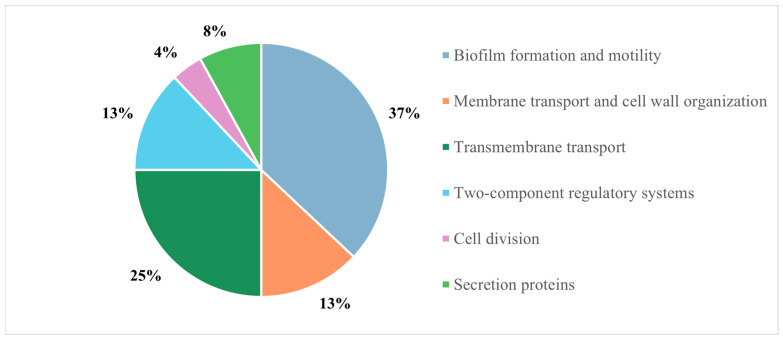
Distribution of Esc(1-21)-1c putative protein partners identified in the pull-down experiment.

A biotinylated form of Esc(1-21)-1c was first immobilized onto avidin-conjugated agarose beads, and the resulting system was incubated with the entire protein extract from P. aeruginosa cultures. The mixture of bacterial proteins was previously incubated with avidin-conjugated agarose beads in the absence of the peptide to prevent non-specific binding, and the proteins not retained on the free beads were then incubated with the peptide bait. Proteins from both the sample and the control were eluted and digested with trypsin on S-Trap™ micro-spin columns, and the resulting peptide mixtures were analyzed by LC-MS/MS. The proteins unspecifically bound to the free agarose beads were used as control. The acquired data were processed using Mascotwebsite www.matrixscience.com, leading to the identification of 245 proteins. Only the proteins identified in the sample were considered as putative Esc(1-21)-1c interactors, whereas those found both in the sample and control were discarded. The putative interactors were then distributed according to the biological processes they are involved in, as defined by the UniProt database (Figure 1).

Among the identified proteins, several members of two-component systems (TCSs) which play pivotal roles in signal transduction pathways in *P. aeruginosa* were found. TCSs are regulatory systems consisting of a response regulator (RR) and a sensor Histidine Kynase (HK), which are able to respond to environmental changes. The identified proteins belonging to TCSs are listed in Table 1. Identification of several RRs acting as transcriptional modulators confirmed previous data suggesting that Esc(1-21)-1c might exert its inhibition mechanism of the MexAB–OprM efflux pump at the transcriptional level.

In fact, in a previous study, we demonstrated that Esc(1-21)-1c interferes with the pump by modulating its expression, and in this work we aimed to dissect the molecular mechanism underlying this inhibitory effect [15]. A thorough investigation into the putative role of the identified response regulators was carried out, and our attention was particularly focused on protein Q9I5H3. In *P. aeruginosa* this protein emerged as an RR belonging to a TCS, with its cognate HK being the sensor protein QseC (UniProt Code Q9I5H2). However, limited information on the specific role of these proteins was available. Sequence homology studies revealed a high degree of homology of Q9I5H3 with *E. coli* QseB protein (UniProt Code P52076), an RR that activates the expression of the *E. coli* AcrAB–TolC efflux pump. Interestingly, the AcrAB–TolC efflux pump was reported to be homologous to the *P. aeruginosa* MexAB–OprM efflux pump [19].

This comparative analysis indicated potential conserved roles for the two homologous RRs in *E. coli* and *P. aeruginosa*, suggesting that Q9I5H3 might be the transcriptional activator of the MexAB–OprM operon. According to this hypothesis, the interaction between Esc(1-21)-1c and Q9I5H3 might then negatively regulate the expression of the MexAB–OprM efflux pump.

### 2.2. Molecular Docking Analysis

Functional proteomics experiments suggested that a specific interaction might occur between the Esc(1-21)-1c peptide and the response regulator Q9I5H3. We were then stimulated to evaluate in silico both the interaction of the peptide with Q9I5H3 and the putative binding of this RR with the promoter region of MexAB–OprM operon by molecular docking analyses.

As it is well known that RRs bind to DNA following dimerization [20], the Q9I5H3 dimer was firstly built through dimerization of two Q9I5H3 subunits by molecular docking (see Section 4 for details). Several hydrophobic interactions, as well as two hydrogen bonds between Phe103 and Glu104 of both subunits were found at the interface between the two Q9I5H3 subunits, suggesting that dimerization might lead to a thermodynamically stable form (Supplementary Figure S1).

**Figure 2:**
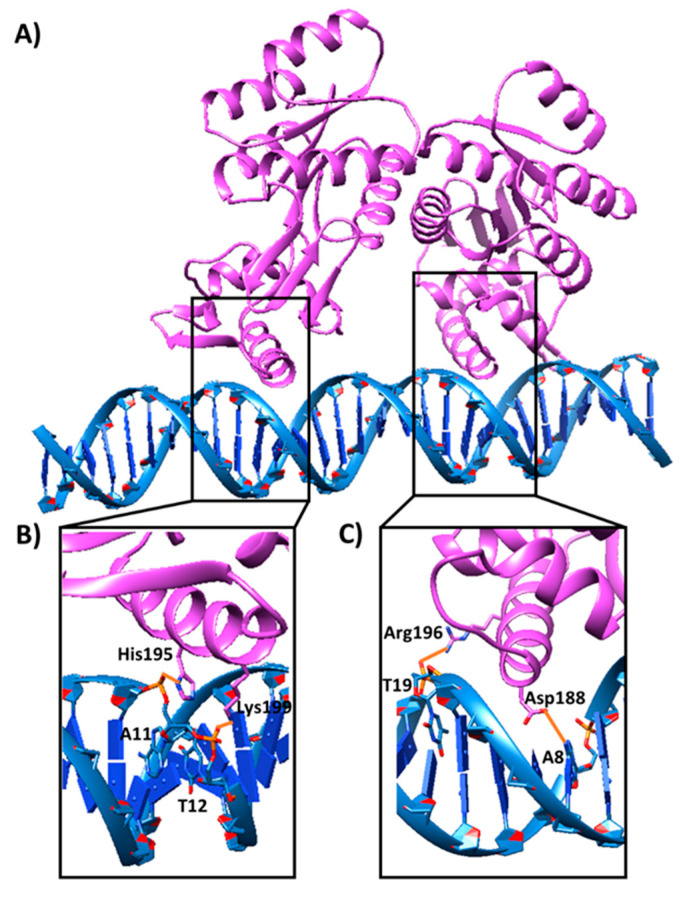
(**A**) Binding mode of the Q9I5H3 dimer when docked to the DNA duplex including the binding consensus site of sequence 5′-GTAAACCTAATGTAAA-3′. The protein dimer and the DNA duplex are shown as purple and blue ribbons, respectively. (**B**,**C**) Enlargements of the protein binding sites on the DNA duplex, which highlight the interactions between the alpha helices of the protein dimer and the major groove of the DNA duplex. Electrostatic interactions/hydrogen bonds are shown as orange bold lines. Amino acids and nucleotides involved in the interactions are labeled and depicted as sticks. Hydrogen atoms are not depicted for ease of illustration.

Then, the putative interaction of the Q9I5H3 dimer with the promoter region of the MexAB–OprM operon was investigated [21]. The docking simulation was carried out using a DNA double helix comprising the MexA promoter (i.e., 5′-GTAAACCTAATGTAAA-3′) and the Q9I5H3 dimer. The analysis returned a negative score of −39.327, indicative of a favourable interaction between the protein dimer and the DNA duplex (Figure 2). Particularly, the alpha helices of the protein dimer align with the major groove of the DNA duplex (Figure 2A), as usually occurs for transcriptional factors [22]. Additionally, the amino acids involved in mediating these interactions, i.e., residues 123–220, belong to a well-characterized DNA-binding domain within the protein sequence reported in the UniProt database for Q9I5H3 (https://www.uniprot.org/uniprotkb/Q9I5H3/entry, accessed on 24 January 2024). In detail, several hydrogen bonds/electrostatic interactions were found between the protein alpha helices, and the DNA grooves including the protonated amino group of Lys199 of one Q9I5H3 subunit and the phosphate group of T12, as well as the imidazole NH of His195 of one Q9I5H3 subunit and the phosphate group of A11, as shown in Figure 2B. Moreover, Figure 2C highlights the interaction between the guanidinium group of Arg196 of the cognate Q9I5H3 subunit and the phosphate group of T19, and the carboxylate group of Asp188 of the cognate Q9I5H3 subunit with the exocyclic amino group of A8.

**Figure 3:**
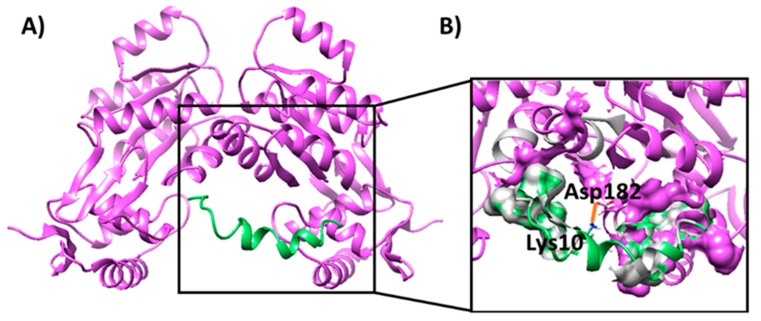
(**A**) Binding mode of the Esc(1-21)-1c peptide when docked to the Q9I5H3 dimer. The protein dimer and the peptide are shown as purple and green ribbons, respectively. (**B**) Enlargement of the peptide binding site on the protein dimer, highlighting their interactions. Electrostatic interactions are shown as orange bold lines, while the hydrophobicity surface is highlighted for residues involved in hydrophobic interactions. Amino acids involved in electrostatic interaction are labeled and depicted as sticks.

Finally, the interaction between the Esc(1-21)-1c peptide and the Q9I5H3 dimer was also evaluated by molecular docking (Figure 3A). Docking data predicted that the peptide might form a stable complex with the Q9I5H3 with a K_d_ value of 5.3 × 10^−6^ M (as predicted by PRODIGY). Several hydrophobic interactions, as well as an electrostatic interaction between Asp182 of the protein dimer and Lys10 of the peptide, were found at the protein–peptide interface contributing to the stability of the complex (Figure 3B and Supplementary Figure S2). Notably, the peptide interacted with a protein region which was located in close proximity to the DNA-binding site on the protein (Figure 2A).

Overall, docking studies predicted that the Q9I5H3 dimer should be able to bind to the promoter sequence of the MexAB–OprM operon, confirming a possible role of the protein as a transcriptional regulator. Moreover, the Esc(1-21)-1c peptide was predicted to form a stable complex with the Q9I5H3 protein validating previous proteomic data. Finally, the AMP might interact with the DNA-binding site of the transcriptional regulator Q9I5H3, possibly preventing its interaction with the MexA promoter and impairing the transcriptional activation of the MexAB–OprM operon.

### 2.3. Recombinant Production and Purification of Q9I5H3 in E. coli

**Figure 4:**
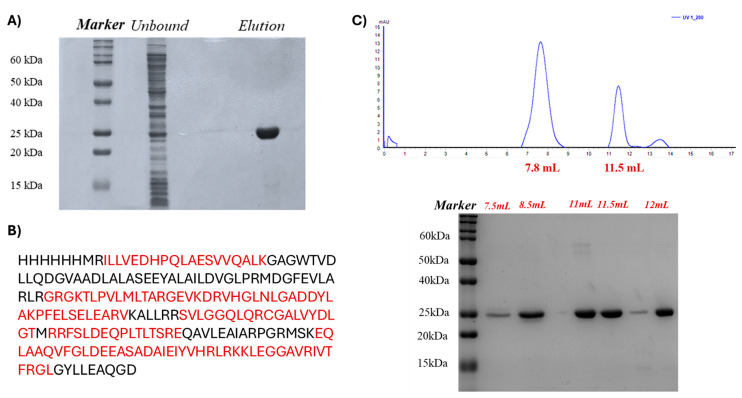
(**A**) SDS-PAGE of protein Q915H3 purified by IMAC affinity chromatography. (**B**) Primary structure validated by peptide mapping using MALDI mass spectrometry. Red-highlighted sequences indicate the peptide map obtained by matching experimental masses with the theoretical molecular weight of Q9I5H3 tryptic peptides). (**C**) Gel filtration chromatogram of the transcriptional regulator Q9I5H3, which reports the absorbance at 280 nm as a function of the elution volume (mL). SDS-PAGE gel of the eluted fractions by molecular exclusion chromatography revealed the expected electrophoretic mobility.

The molecular docking predicted specific interactions between Esc(1-21)-1c and Q9I5H3, which were experimentally investigated by fluorescence spectroscopy and EMSA analyses by using a recombinant form of the *P. aeruginosa* protein. The *P. aeruginosa* PA0756 gene corresponding to the Q9I5H3 sequence fused to a N-terminal 6-histidine tag was cloned and expressed in *E. coli* BL21 cells, resulting in the production of the recombinant protein in a large amount. The protein was purified to homogeneity by immobilized metal ion affinity chromatography (IMAC) on with precharged Ni Sepharose™ High Performance. The purity of the protein was assessed by SDS-PAGE and its primary structure validated by peptide mapping using MALDI mass spectrometry following triptic digest and direct mass spectrometric analysis of the resulting peptide mixture (Figure 4A,B and Supplementary Figure S3).

The quaternary structure of the protein was assessed in solution by gel filtration chromatography on a Superdex^®^ 75 column previously calibrated with a mixture of appropriate standard proteins. The calibration curve yielded an R^2^ value of 0.992 and was then used for further analyses. The purified Q9I5H3 protein was subjected to gel filtration chromatography, revealing two elution peaks at a volume of 7.8 mL and 11.5 mL, respectively, as shown in Figure 4C. Data processing from the calibration curve indicated an apparent molecular weight of approximately 50 kDa and 25 kDa, respectively, demonstrating that the protein exists in both monomeric and dimeric forms in solution. As the regulator has intrinsic dimerization domains that promote self-association, the expression conditions used to synthesized Q9I5H3 in *E. coli* resulted in the production of a recombinant protein very similar to the *P. aeruginosa* counterpart that maintained its natural propensity for dimerization. In *P. aeruginosa*, dimerization of Q9I5H3 is necessary for its transcriptional regulator activity within the TCS system, as it facilitates DNA-binding, increases protein stability, and enables regulatory interactions.

### 2.4. Fluorescence Binding Experiments Between Esc(1-21)-1c and Q9I5H3

**Figure 5:**
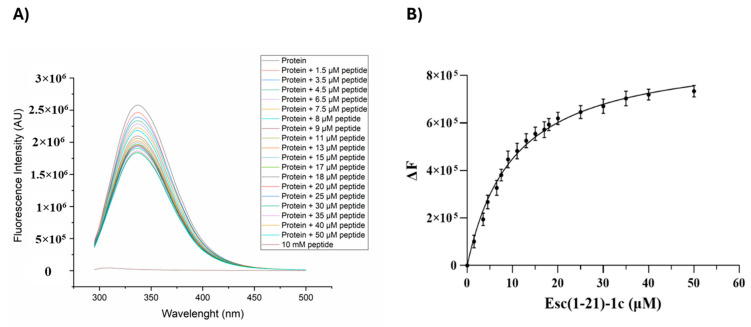
(**A**) Fluorescence spectra (λexc = 280 nm) of Q915H3 obtained by adding the indicated concentrations of Esc (1-21)-1c. (**B**) Binding curve related to the interaction between Esc(1-21)-1c and Q915H3 as determined by fluorescence experiments.

The interaction of the Esc(1-21)-1c peptide with Q9I5H3 was experimentally evaluated by fluorescence-based binding experiments. The dimeric form of the Q9I5H3 protein, purified by gel filtration, was titrated with increasing concentrations of Esc(1-21)-1c, and changes in the fluorescence emission of the protein were monitored (Figure 5). The high number of aromatic residues in Q9I5H3 resulted in a significant fluorescence emission intensity at about 340 nm, eliminating the need for specific fluorescence tags or labels. The fluorescence intensity of Q9I5H3 significantly decreased with increasing concentration of the peptide, confirming peptide–protein interaction. Analysis of the fluorescence data allowed us to calculate the dissociation constant of the complex, which had a value of 10 ± 0.98 µM. This result is consistent with docking calculations, confirming the formation of a stable protein–peptide complex and indicating a good affinity of Esc(1-21)-1c for Q9I5H3.

### 2.5. EMSA-Based Binding Experiments Between Q9I5H3 and Duplex DNA

**Figure 6:**
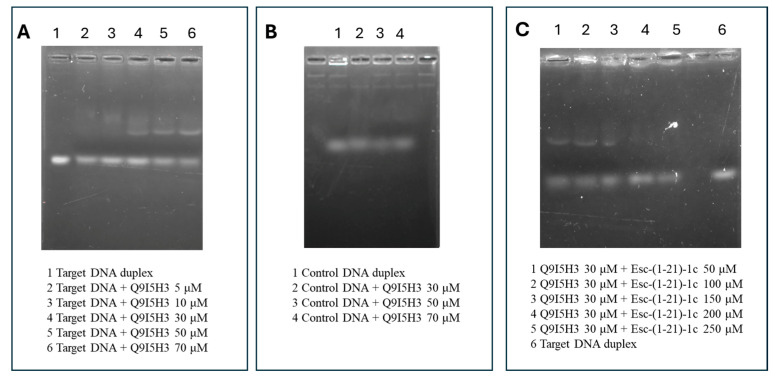
EMSA performed on Q9I5H3 proteins. (**A**) Lane 1, target DNA duplex. Lanes 2 to 6 target DNA duplex incubated with increasing amounts of Q9I5H3 (from 5 to 70 μM). (**B**) Lane 1, control DNA duplex. Lanes 2 to 4 control DNA duplex incubated with increasing amounts of Q9I5H3 (from 30 to 70 μM). (**C**) Lanes 1 to 5, 30 μM of Q9I5H3 protein first incubated with increasing concentration of Esc(1-21)-1c (from 50 to 250 μM), followed by target DNA duplex addition. Lane 6, target DNA duplex.

Finally, the DNA-binding ability of the recombinant Q9I5H3 protein was investigated by EMSA analysis. The DNA duplex featured by the target promoter sequence was incubated with increasing concentrations of Q9I5H3. The resulting DNA–protein complexes were resolved on a 1.5% agarose gel and visualized by UV transillumination. As shown in Figure 6A, the occurrence of a lower mobility band was observed, of which intensity increased following incubation with increasing concentration of the protein. This effect was confirmed by the concomitant decrease in the intensity of the free DNA probe band in a dose-dependent manner, supporting the formation of stable DNA–protein complex. Crucially, when the same experiment was performed using a non-specific DNA sequence (Figure 6B), no retardation shift was detected, even at the highest protein concentrations. This result strongly demonstrates that Q9I5H3 selectively binds to its cognate promoter sequence and not random DNA, confirming the specificity of the interaction.

The putative effect of the Esc(1-21)-1c peptide on the Q9I5H3–DNA interaction was investigated by a competition assay in which the recombinant Q9I5H3 protein was pre-incubated with increasing concentrations of the peptide before adding the DNA probe. The protein–DNA complexes in the presence of different concentrations of Esc(1-21)-1c were again examined by EMSA. Figure 6C clearly shows a progressive reduction in the intensity of the shifted band, corresponding to the Q9I5H3–DNA complex at increasing concentration of peptide, that completely disappeared in the presence of 200µM peptide. This result confirms that Esc(1-21)-1c can affect the interaction of Q9I5H3 with the promoter of MexAB–OprM operon altering the transcription of target genes.

## 3. Discussion

AMPs are emerging as a class of multiaction molecules in the challenging context of antibiotic resistance. These compounds are considered potential new antimicrobials both for their direct action in killing pathogens and also as compounds capable of exerting an indirect effect on the microorganism when used at concentrations lower than the active dose. Previous studies investigating the global effects of sub-lethal doses of the AMP Esc(1-21)-1c on *P. aeruginosa* revealed an effective downregulation of the MexA–MexB–OprM efflux pump [14]. However, the precise molecular mechanism underlying this effect remained undefined. This study aimed to elucidate the specific mode of action of Esc(1-21)-1c by employing a functional proteomic approach to identify putative specific protein interactors and characterize their functional roles in efflux pump regulation. A pull-down experiment using a biotinylated form of the peptide allowed us to identify several putative interactors, among which a number of factors belonging to two-component regulatory systems (TCSs) were found.

### 3.1. Mechanistic Interpretation: Role of Two-Component Systems in Bacterial Regulation

This finding is particularly significant given the central role of TCS in bacterial gene regulation and adaptive responses. TCS represent a typical regulatory system in bacteria comprising a response regulator (RR) and a sensor histidine kinase (HK). Upon environmental changes, the HK undergoes autophosphorylation at a conserved histidine residue, transferring the phosphoryl group to the cognate RR [23]. The RR protein generally consists of two domains: a receiver domain (RD) that accepts the phosphoryl group and an effector domain (ED) that mediates the regulatory response. Following phosphorylation of the RD, the ED becomes activated, inducing an allosteric conformational change in the RR that facilitates homodimerization and enables DNA promoter binding with increasing affinity, thus initiating the transcription of target genes [21,24,25,26,27,28,29,30,31,32,33,34,35,36].

### 3.2. Q9I5H3 as a Novel Transcriptional Regulator of MexAB–OprM

Sequence homology analyses carried out on the identified RRs indicated a significant similarity of the Q9I5H3 protein with the homologous QseB in *E. coli*. QseB is the response regulator of the QseBC two-component system that affects antibiotic sensitivity in *E.coli* by regulating the transcription of the acrAB–tolC operon [27]. This operon activates the expression of the *E. coli* AcrAB–TolC efflux pump that was reported to be homologous to the *P. aeruginosa* MexAB–OprM complex [19]. This observation suggested Q9I5H3 as a transcriptional activator of the MexAB–OprM operon, representing a novel regulatory mechanism previously uncharacterized in *P. aeruginosa*. Despite its apparent relevance, limited information was available about the specific functions of the Q9I5H3 response regulator. Thus, we employed molecular modeling and experimental validation approaches to deeply investigate the functional role of this transcription factor. Molecular docking predictions suggested that this protein might interact with the promoter region of the MexAB–OprM operon in its dimeric form, consistent with the typical DNA-binding mechanism of transcriptional regulators. Moreover, the Esc(1-21)-1c peptide was predicted to form a stable complex with the Q9I5H3 response regulator, with the peptide binding site located close to the protein’s DNA-binding domain. These predictions were experimentally confirmed by fluorescence-based binding experiments and electrophoretic mobility shift assays using a recombinant form of the protein.

### 3.3. Therapeutic Implications of Targeting Q9I5H3

MexAB–OprM is considered the main contributor to antibiotic resistance in *P. aeruginosa* [15] as this multidrug efflux pump is able to export several classes of antibiotics. The expression of this operon is mainly controlled by the repressor genes MexR [28], nalC [29], and nalD. The use of antibiotics selects *P. aeruginosa* strains with increased MexAB–OprM expression [30,31] essentially due to several mutations occurring in repressor genes. The key role played in *P. aeruginosa* antibiotic resistance highlights this pump as a promising clinical target, stimulating intense research for the discovery of specific inhibitors. Moreover, several investigations demonstrated that these multidrug transporters are able to export a broad range of ligands outside the cells, including their own inhibitors. In this respect, the identification of Q9I5H3 as a new essential regulator of the MexAB–OprM efflux pump suggests this factor as a possible target for the development of new specific drugs.

### 3.4. Summary of Findings

In conclusion, this study provides a detailed description of the mechanism of action of Esc(1-21)-1c and highlights the critical role of Q9I5H3 as a new transcriptional regulator of the MexAB–OprM operon. In particular, the peptide appears to interfere with the activation of the MexAB–OprM efflux pump via direct interaction with the Q9I5H3 response regulator. Inhibition of Q9I5H3 would lead to the downregulation of the multidrug transporter, making *P. aeruginosa* sensitive to conventional antibiotics, as already reported [14]. Finally, these findings point to Q9I5H3 as a possible specific target for the rational design of new inhibitors that might open the way to new therapeutic strategies in synergy with conventional antibiotic treatments.

## 4. Materials and Methods

### 4.1. Peptide Synthesis

Biotinylated Esc(1-21)-1c was synthetically produced by Bio-Fab Research (Rome, Italy) using a standard F-moc protocol.

### 4.2. Bacterial Growths

The reference strain *P. aeruginosa* PAO1 was used in this study, as previously reported [14]. For the functional proteomics analysis, a pre-inoculum of the bacterium was made in Luria–Bertani (LB) and incubated overnight at 37 °C. The next day, a fresh inoculum was made in LB and incubated at 37 °C with shaking (180 rpm) until an optical density (O.D.) of 0.5 was reached (λ = 590 nm) and then was incubated for a further 3 h, following the treatment time previously reported [14]. The microbial culture was then divided into 50 mL tubs (~25 mL per tube) and centrifuged for 20 min at 5000 rpm at 4 °C in a refrigerated centrifuge. The pellets were stored at −20 °C until they were used.

### 4.3. Functional Proteomics Experiment

An amount of 200 μL of dry avidin-conjugated agarose beads were incubated with a solution containing 2 mg/mL of biotinylated Esc(1-21)-1c for 30 min at 4 °C under stirring. The supernatant was removed by centrifugation for 10 min at 3000 rpm at 4 °C. *P. aeruginosa* cell pellet was recovered by centrifugation at 4 °C for 20 min at 5000 rpm and resuspended in 5 mL of Cell Lysis Buffer (20 mM Tris-HCl pH 8.0, 500 mM NaCl, 4 mM dithiothreitol, 1 mM phenylmethanesulfonyl fluoride solution, cOmpleteTM protease inhibitor). Following mechanical lysis, the cell debris was removed, and the supernatant was recovered and quantified by Bicinchoninic acid (BCA) assay, following the manufacturer’s instructions (Thermo Scientific, Rockford, IL, USA).

An amount of 2.5 mg of proteins was incubated on free agarose beads for 2 h at 4 °C under stirring to remove possible non-specific binding, according to the pre-cleaning procedure. The supernatant containing the unbound membrane proteins was recovered by centrifugation for 10 min at 3000 rpm at 4 °C and then incubated on agarose beads with the immobilized peptide for 3 h at 4 °C under stirring. The beads were washed with 5 volumes of phosphate-buffered saline (PBS), and the peptide-interacting proteins were released by competitive elution with 500 μL of PBS supplemented with 2 mM biotin for 1 h at 4 °C under stirring. The eluted proteins were quantified by BCA assay. An amount of 50 µg of eluted proteins underwent trypsin digestion on S-trap^TM^ micro-spin columns, following the manufacturer’s instructions (Protifi, Huntington, NY, USA). Hydrolyzed peptide mixtures were analyzed by nano LC-MS/MS on a LTQ Orbitrap XL coupled to a nanoLC system (ThermoFisher Scientific, Waltham, MA, USA). Peptide fractionation was performed onto a C18 capillary reverse-phase column (200 mm, 75 µm, 5 µm) operating at a flow rate of 250 nL/min, using a step gradient of eluent B (0.2% formic acid, 95% acetonitrile) and a non-linear 5% to 50% gradient of eluent B (95% acetonitrile, 0.2% formic acid) over 260 min. Mass analyses were performed in data-dependent acquisition (DDA) mode by fragmenting the 10 most intense ions in the CID modality.

The obtained data were used to search a non-redundant protein database using an in-house version of the Mascot software, leading to the identification of putative Esc(1-21)-1c protein interactors. The parameters used for protein identification are listed in Table 2.

### 4.4. Molecular Docking

The PDB file for the protein with UniProt code Q9I5H3 was obtained from the AlphaFold Protein Structure Database [32,33], the NMR structure of Esc(1-21)-1c peptide of sequence GIFSKLAGKKIKN^d^LLI^d^SGLKG was kindly provided by Ghosh et al. [34], while the PDB structure of the DNA duplex, comprising the MexA promoter sequence (i.e., 5′-GTAAACCTAATGTAAA-3′) [21] as well as its complementary strand, was built with the aid of UCSF Chimera [35]. Molecular docking calculations were carried out using pyDockWEB [36] for protein–protein and protein–peptide systems and pyDockDNA [37] for protein–DNA systems. Docking poses were ranked based on their score and the ones with the most negative score values were selected for further analysis. Specifically, molecular docking calculations to obtain the structural model of the Q9I5H3 homodimer were performed, including distance restraints between residues 2–116, based on previous knowledge of the dimerization domains of these TCS [26] and information reported in the UniProt database for Q9I5H3 (https://www.uniprot.org/uniprotkb/Q9I5H3/entry, accessed on 24 January 2024). Finally, the docking pose featured by the most negative score value and proper symmetry requirements was selected [38]. Interactions were evaluated by LigPlot+ [39], while dissociation constants were estimated by PRODIGY [40]. Molecular modeling figures were drawn by UCSF Chimera and LigPlot+.

### 4.5. Recombinant Production and Purification of Protein Q9I5H3

The PA0756 gene of *P. aeruginosa*, encoding the putative Esc(1-21)-1c interactor, was cloned into the pET28a vector containing the coding sequence for the corresponding recombinant protein (UniProt code Q9I5H3) fused to a 6-histidine tag at the N-terminus. Expression of the recombinant protein was carried out in BL21 *E. coli* cells, in LB medium at 37 °C with 100 µg/mL kanamicin, and the over-expression of Q9I5H3 was induced in the exponential phase with isopropyl-thio-β-d-galactoside (IPTG) at a final concentration of 1 mM. Cultures were grown at 37 °C for 3 h and cells were then retrieved by centrifugation at 5000 rpm for 20 min at 4 °C. The cellular pellet was resuspended into 0.1 M Na_2_HPO_4_, 0.15 M NaCl, 1 mM PMSF, pH 7.4. Cells were lysed by sonication for 20 min, and the sample was centrifuged at 13,000 rpm for 30 min at 4 °C, allowing the separation of the insoluble fraction, which contained inclusion bodies from the soluble sample. Proteins were purified from the soluble fraction. His6-Q9I5H3 was purified by affinity chromatography on His-Trap^TM^ FF 1mL column (Cytiva, Uppsala, Sweden) and eluted with 500 mM imidazole in 20 mM Na_2_HPO_4_, 0.5 M NaCl, pH 7.4, performing a step gradient with 100 mM imidazole for 20 column volumes (CV), followed by 250 mM imidazole for 20 CV, and 500 mM imidazole for 5 CV.

Protein concentration was determined with the Bradford Reagent from Sigma Aldrich Merck, St. Louis, MO, USA), using BSA as a standard. The primary structure of Q9I5H3 was validated by mass mapping using MALDI-TOF on a MALDI Voyager-DET STR spectrometer (Applied Biosystems, CA, USA). Following electrophoresis and staining, the gel band corresponding to the molecular weight of Q9I5H3 was excised and subjected to in situ hydrolysis. Reduction was achieved using 10 mM DTT in 50 mM AMBIC (Sigma–Aldrich Merck, St. Louis, MO, USA) for 45 min at 56 °C to reduce cysteine residues involved in disulfide bridges. The gel band was dehydrated with 100% ACN and then rehydrated with 50 mM AMBIC, containing 55 mM IAM for the alkylation reaction of cysteine residues in a dark environment at room temperature for 30 min. The dehydrated gel band was treated with 10 ng·μL^−1^ trypsin (Sigma–Aldrich Merck, St. Louis, MO, USA) solution in 50 mM AMBIC pH 8.0 for 1 h at 4 °C. The sample was then incubated for 16 h at 37 °C in 50 mM AMBIC. Following hydrolysis, the supernatant was collected, acidified with 20% TFA, (Sigma–Aldrich Merck, St. Louis, MO, USA), and any remaining peptides in the gel were extracted with 50 μL of ACN. A second extraction was performed using 20 μL of 0.2% formic acid (HCOOH, Chem-Lab, Eernegem, Belgium) followed by ACN. The obtained peptide mixture was dried by a Speed-Vac system and resuspended in 0.2% HCOOH.

The sample was co-crystallized on a MALDI plate together with 10 mg/mL of α-cyano-4-hydroxycinnamic acid dissolved in 70% ACN and ionized by laser pulse. The analysis was performed positively, using the instrument in reflectron mode (range of 400–500 *m*/*z*).

Finally, the quaternary structure of Q9I5H3 was assessed by gel filtration chromatography on Superdex^®^ 75 10/300 GL eluted with 50 mM Tris-HCl pH 8.0 containing 150 mM NaCl.

### 4.6. Binding Experiment by Fluorescence Spectroscopy

Fluorescence experiments were performed on a Fluoromax-4 spectrofluorometer from Horiba Scientific, Piscataway, NJ, USA, using 1 cm optical path-length quartz cell under controlled temperature conditions (Peltier control system at 20 °C). Titrations were carried out in 50 mM Tris-HCl, pH 8.0 and 150 mM NaCl. Excitation wavelength used for the fluorescence titrations was 280 nm, and the spectra were recorded in the range 300–500 nm (slit 3 nm). Titrations were carried out at a fixed concentration (35 μM) of Q9I5H3 by adding increasing amounts of Esc(1-21)-1c peptide (from 1.5 to 50 µM), and the fluorescence emission was monitored at 337 nm. The experiments were performed in duplicate. The change in the fluorescence intensity was reported as a function of the peptide concentration and the data fitted with the “one site specific binding” equation of GraphPad Prism 9 (GraphPad Software LLC, La Jolla, CA, USA).

### 4.7. Protein–DNA Interaction by Electrophoretic Mobility Shift Assay (EMSA)

The interaction of Q9I5H3 protein with DNA was investigated by EMSA experiments using the target DNA duplex as well as a control duplex-forming sequence generally used as a model of standard B-DNA duplex [41], the so-called Dickerson dodecamer (Supplementary Table S1). Sense and antisense oligonucleotides were annealed by incubation at 95 °C for 5 min and then gradual cooling to room temperature. Different concentrations of proteins were incubated with the DNA duplexes (0.05 µM) for 30 min at 25 °C in 20 µL of 25 mM HEPES pH 7.6, containing 50 mM KCl, 12.5 mM MgCl2, 1 mM DTT, 20% glycerol, 0.1% Triton.

For the assays, 30 μM of protein was pre-incubated with increasing concentrations of Esc(1-21)-1c for 10 min at 25 °C. Following this pre-incubation, the DNA duplex was added, and the mixture was incubated for additional 20 minutes under standard EMSA conditions.

The maximum concentration of protein (70 μM) incubated with control DNA duplex was used as negative control.

All samples were separated on 1.5% agarose gel in Tris-borate-EDTA (TBE) running buffer at room temperature, using GelRed as dye. Visualization of DNA samples was carried out using UV radiation.

## Figures and Tables

**Table 1:** List of proteins belonging to TCS identified in the pull-down experiment.

UniProt Code	Protein Name	Gene	Peptides	Biological Function
Q9I2U3	Two-component response regulator ParR	*parR*	3(1)	Two-component regulatory system
Q9HU59	DNA-binding transcriptional regulator NtrC	*ntrC*	3(1)	Regulation of biofilm formation
Q9HV31	Sensor protein kinase PmrB	*pmrB*	4(1)	Two-component regulatory system
G3XCT6	Chemotaxis protein CheA	*PA1458*	1(1)	Chemotaxis
Q9HX42	histidine kinase	*ladS*	3(1)	Biofilm process
Q9HYE4	histidine kinase	*PA3462*	2(1)	Sensor kinase activity
Q9I5H3	Probable two-component response regulator	*PA0756*	5(2)	Two-component regulatory system
Q9HWI4	Histidine kinase	*bfiS*	4(1)	Biofilm formation
P24908	Putative transcriptional regulator	*PA0034*	7(1)	Two-component regulatory system
Q9I6K5	EAL domain-containing protein	*PA0285*	4(1)	Regulation of DNA-templated transcription

**Table 2:** Selected parameters for LC-MS/MS analysis.

Parameters	
Fixed Modifications	Carbamidomethyl (C)
Variable Modifications	Oxidation (M) Gln-> pyro-Glu (N-Term Q)
Enzyme	Trypsin
Max Missed Cleavages	3
Minimum Number of Peptides	5
False Discovery Rate (FDR)	0.01

## Data Availability

The raw data presented in the study are openly available in https://doi.org/10.6084/m9.figshare.30075721.

## Supplementary Materials

The following supporting information can be downloaded at https://www.mdpi.com/article/10.3390/ijms26209940/s1.
