# Supplementary material for: The Esc(1-21)-1c Antimicrobial Peptide Inhibits a Specific Transcriptional Activator of the MexAB–OprM Efflux Pump in P. aeruginosa
Source: Int J Mol Sci. 2025 Oct 13;26(20):9940. doi: 10.3390/ijms26209940 (PMC12563431; doi:10.3390/ijms26209940)
Supplement: Supplementary file 1 [file ijms-26-09940-s001.zip › ijms-3891506-supplementary.pdf]

Supplementary Table S1

The target DNA sequence used in the EMSA experiments

**FW**	**5'CGAGTAAACCTAATGTAAATGTGGTTGATC 3'**
**RW**	**5'GCTCATTTGGATTACATTTACACCAACTAG 3'**

Figure S1

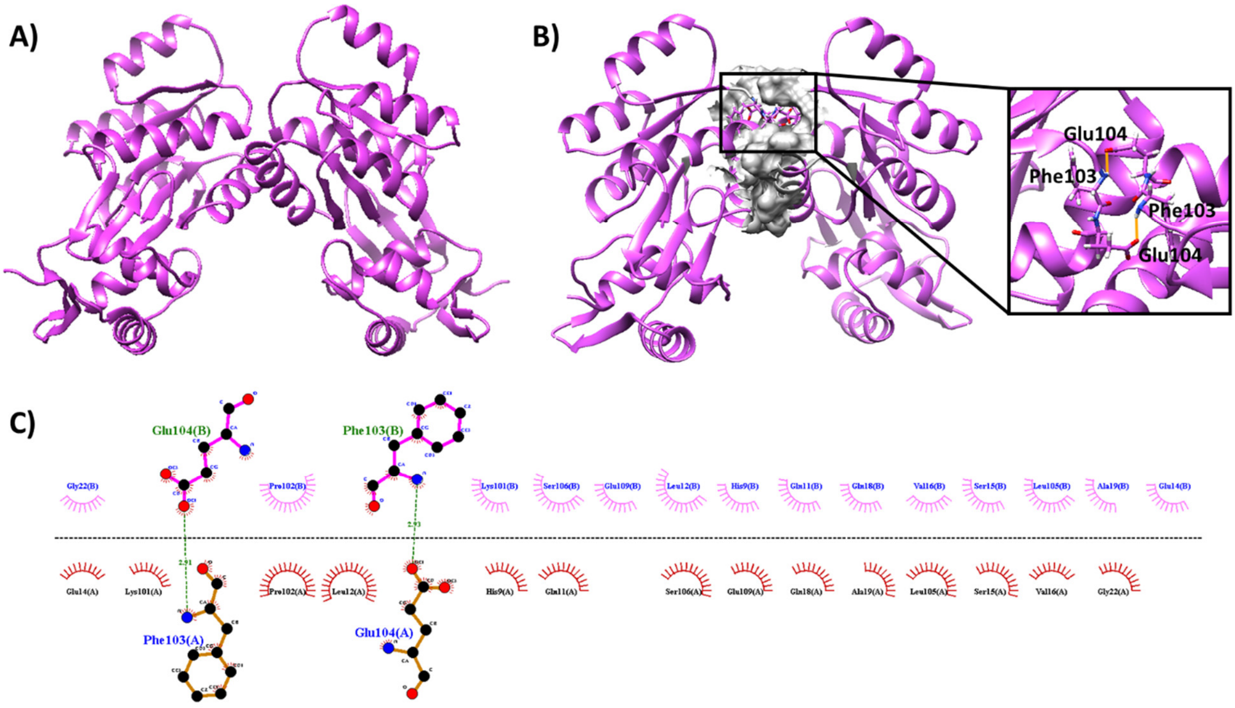

Figure S1. A) Binding mode of a Q9I5H3 subunit when docked to an additional Q9I5H3 subunit. The protein dimer is shown as purple ribbon. B) Enlargement of the protein-protein interface region, which highlights the interactions between the two Q9I5H3 subunits. Hydrogen bonds are shown as orange bold lines, while the hydrophobicity surface is highlighted for residues involved in hydrophobic interactions. Amino acids involved in the interactions are labeled and depicted as sticks. C) 2D interaction map. C, N and O atoms are reported in black, blue and red, respectively. Hydrogen atoms are not depicted for ease of illustration. Hydrogen bonds and hydrophobic contacts are depicted as green dashed lines and red/purple arcs with radiating lines, respectively. Amino acids involved in the interactions are labeled.

Figure S2

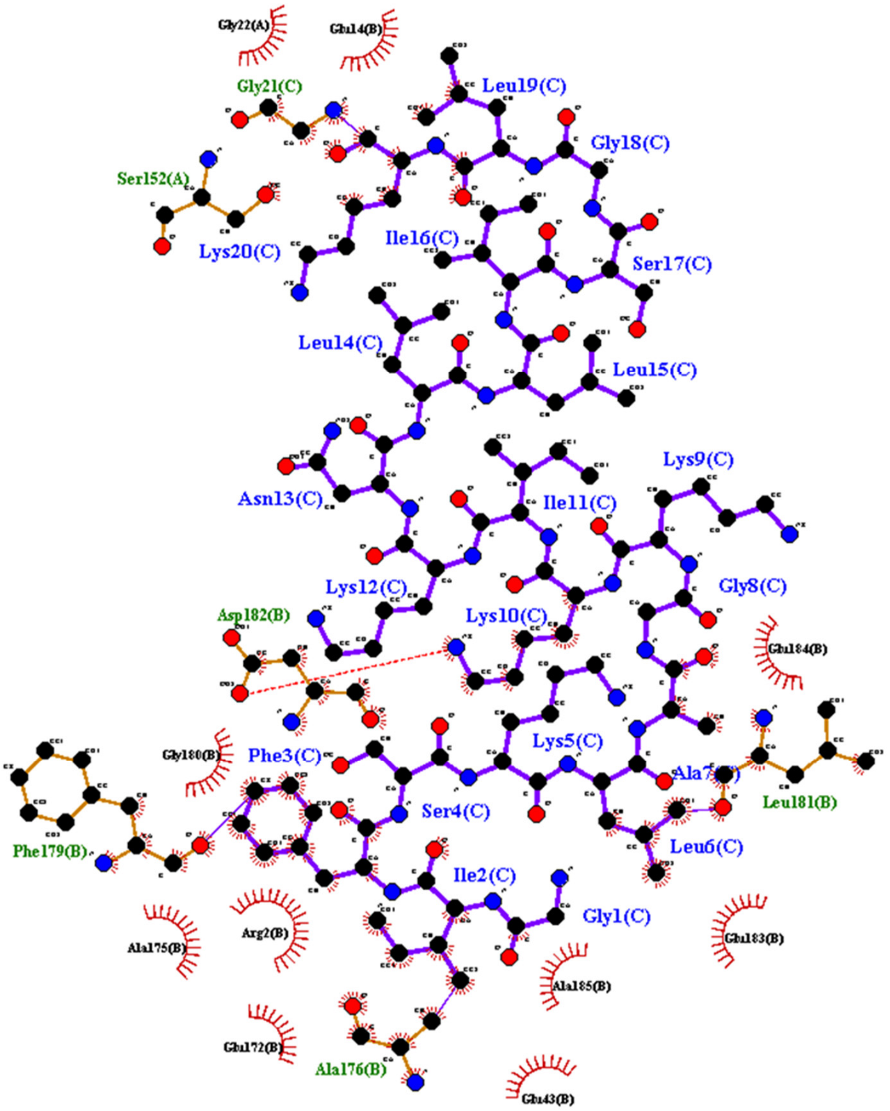

Figure S2. 2D map of the interactions for the Esc(1-21)-1c peptide when docked to the Q9I5H3 dimer. C, N and O atoms are reported in black, blue and red, respectively. Hydrogen atoms are not depicted for ease of illustration. Electrostatic interactions and hydrophobic contacts are depicted as red dashed lines and red arcs with radiating lines, respectively. Amino acids involved in the interactions are labeled.

Figure S3

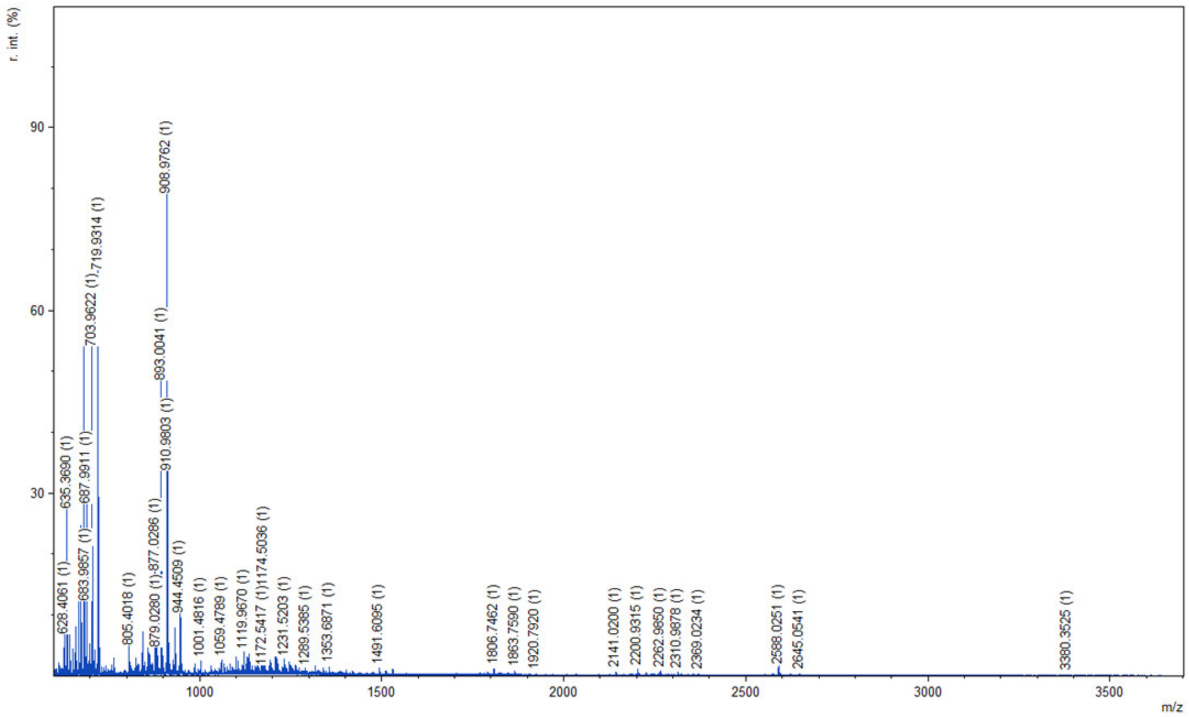

Figure S3 Mass spectrum obtained to determine the primary structure of the transcriptional regulator Q9I5H3. The spectrum shows the relative abundance of ions as a function of the mass/charge (m/z) ratio.
